# Therapeutic Potential of a Prolyl Hydroxylase Inhibitor FG-4592 for Parkinson’s Diseases *in Vitro* and *in Vivo*: Regulation of Redox Biology and Mitochondrial Function

**DOI:** 10.3389/fnagi.2018.00121

**Published:** 2018-04-27

**Authors:** Xuan Li, Xin-Xin Cui, Ya-Jing Chen, Ting-Ting Wu, Huaxi Xu, Huiyong Yin, Yun-Cheng Wu

**Affiliations:** ^1^Department of Neurology, Shanghai General Hospital, Shanghai Jiao Tong University School of Medicine, Shanghai, China; ^2^Neuroscience Initiative, Sanford Burnham Prebys Medical Discovery Institute, La Jolla, CA, United States; ^3^Key Laboratory of Food Safety Research, Shanghai Institutes for Biological Sciences, Chinese Academy of Sciences, Shanghai, China; ^4^Key Laboratory of Food Safety Risk Assessment, Ministry of Health, Beijing, China; ^5^School of Life Sciences and Technology, ShanghaiTech University, Shanghai, China

**Keywords:** Parkinson’s disease, FG-4592, HIF-1α, PGC-1α, mitochondrial function

## Abstract

As the main transcription factor that regulates the cellular responses to hypoxia, Hypoxia-inducible factor-1α (HIF-1α) plays an important role in the pathogenesis of Parkinson’s disease (PD). HIF-1α is normally degraded through ubiquitination after hydroxylation by prolyl hydroxylases (PHD). Emerging evidence has suggested that HIF PHD inhibitors (HIF-PHI) may have neuroprotective effects on PD through increasing HIF-1α levels. However, the therapeutic benefit of HIF-PHI for PD remains poorly explored due to the lack of proper clinical compounds and understanding of the underlying molecular mechanisms. In this study, we examined the therapeutic benefit of a new HIF-PHI, FG-4592, which is currently in phase 3 clinical trials to treat anemia in patients with chronic kidney diseases (CKD) in PD models. FG-4592 attenuates MPP^+^ -induced apoptosis and loss of tyrosine hydroxylase (TH) in SH-SY5Y cells. Pretreatment with FG-4592 mitigates MPP^+^-induced loss of mitochondrial membrane potential (MMP), mitochondrial oxygen consumption rate (OCR), production of reactive oxygen species (ROS) and ATP. Furthermore, FG-4592 counterbalances the oxidative stress through up-regulating nuclear factor erythroid 2 p45-related factor 2 (Nrf-2), heme oxygenase-1 (HO-1) and superoxide dismutase 2 (SOD2). FG-4592 treatment also induces the expression of Peroxisome proliferator-activated receptor-γ coactivator-1α (PGC-1α) through increasing the phosphorylation of AMP-activated protein kinase (AMPK). In MPTP-treated mice, FG-4592 protects against MPTP-induced loss of TH-positive neurons of substantia nigra and attenuates behavioral impairments. Collectively, our study demonstrates that FG-4592 is a promising therapeutic strategy for PD through improving the mitochondrial function under oxidative stress.

## Highlights

(1) FG-4592 attenuates MPP^+^-induced apoptosis in SH-SY5Y cells by induction of HIF-1α.

(2) FG-4592 improves the mitochondria function through HIF-1α-AMPK-PGC-1α pathway and activation of autophagy.

(3) FG-4592 protects the dopaminergic neurons from apoptosis and attenuated the behavioral impairments of MPTP-treated mice.

## Introduction

Parkinson’s disease (PD) is the second major neurodegenerative disease after Alzheimer’s disease and the estimated prevalence of PD in people older than 60 years is 1.0%, and 3.0% in those aged 80 years and older (Tanner and Goldman, 1996). PD is characterized by the loss of neurons and the presence of Lewy bodies that contain aggregates of α-synuclein in the substantia nigra (Pollanen et al., 1993; Spillantini et al., 1997). But the exact pathogenesis leading to the selective dopaminergic cell loss in PD remains poorly defined. Accumulating evidence appears to suggest that mitochondrial dysfunction, oxidative stress, and dys-regulation of autophagy and apoptosis play an important role in the pathogenesis of PD.

It is well-established that severe and prolonged hypoxia contributes to the brain damage (Sharp and Bernaudin, 2004), but exposure to moderate hypoxia alone does not cause neuronal death as long as cerebral blood flow is maintained (Teli and Rajanikant, 2013). Since HIF-1α is the master transcriptional regulator of cellular responses to hypoxia, it may be one major pathway involved in neuroprotection (Ran et al., 2005). Additionally, a body of evidence has also shown that HIF-1α protects against the ischemic stroke (Speer et al., 2013; Zhou et al., 2017). HIF-1α activates a various target genes that regulate angiogenesis, erythropoiesis, energy metabolism, cell proliferation, and cell cycle control (Majmundar et al., 2010). HIF-1α protein is degraded through hydroxylation by PHD, which promotes the interaction between HIF-1α and the pVHL ubiquitin E3 ligase complex (Maxwell et al., 1999; Srinivas et al., 1999; Ohh et al., 2000). The HIF-PH requires iron, 2-oxoglutarate, oxygen, and ascorbate as cofactors (Ivan et al., 2001; Jaakkola et al., 2001). MPTP treatment inhibits HIF-1α accumulation in dopaminergic cell lines PC12 and in mice (Agani et al., 2000), suggesting that the function of HIF-1α is weakend in PD. Previous studies have shown that HIF-PH inhibitors (HIF-PHI) have neuroprotective effects on PD models by increasing HIF-1α expression. DHB and compound A (appear to inhibit PHD by removing the iron cofactor) can also be used as a HIF-PHI protects against MPTP induced nigral dopaminergic cell damage through up-regulating protein levels of HIF-dependent genes HO-1 and MnSOD (Lee et al., 2009). Our laboratory also reported the neuroprotective effects of iron chelators DFO and Orexin-A in MPP^+^ treated SH-SY5Y cell model of PD with its ability to stabilize HIF-1α and activate HIF-dependent genes (Wu et al., 2010; Feng et al., 2014). However, the therapeutic potential of HIF-PHI remains poorly explored due to the lack of proper clinical agents. In recent years there has been a new HIF-PHI FG-4592 (Roxadustat /ASP1517), which is an orally active, novel, potent, and transient small-molecule HIF-PHI. Two Phase II studies conducted in China this year approved the safety and efficacy of FG-4592(Chen et al., 2017). It was also being investigated in global Phase 3 program for the treatment of anemia in patients with chronic‘kidney diseases (CKD) and achieved a good effect (Besarab et al., 2015; Provenzano et al., 2016). Thus, we hypothesized that FG-4592 might protect dopaminergic neurons through upregulation of HIF-1α since some studies have demonstrated it can partially across the blood brain barrier and induced the expression of HIF-1α in brain tissue of mice (Hoppe et al., 2016). In this study, we found that FG-4592 protected against MPP^+^-induced neuronal injury, mitochondria dysfunction, oxidative stress and reduction of autophagy *via* HIF-1α induction *in vitro* and *in vivo*. Our study herein clear demonstrates that FG-4592 is a promising therapeutic agent for PD.

## Materials and Methods

### Cell Culture and Transfection

SH-SY5Y neuroblastoma cells were cultured in Dulbecco’s modified eagle’s medium (DMEM, Hyclone) containing 10% fetal calf serum (Hyclone, Logan City, UT, United States) and grown in a CO_2_ incubator maintained at atmospheric oxygen levels and 5% CO_2_. Dissolved MPP^+^ (Sigma Aldrich, St. Louis, MO, United States) in phosphate buffered saline (PBS) to a storage concentration of 125 mM and MPP^+^ was added to the culture medium to achieve the final concentration. FG-4592 (Selleckchem, Houston, TX, United States) stocks were dissolved in dimethyl sulfoxide (DMSO) at the concentration of 50 mM. Right before each experiment, a stock of FG-4592 was added to the culture medium to achieve the final concentration at 50 μM. Thus 0.1% of DMSO was present in the final culture. They were both stored at -20°C. At MPP^+^ and FG-4592 co-treatment group, FG-4592 was added 6 h before MPP^+^.

SH-SY5Y cells were transiently transfected with siRNA-HIF-1α (against HIF-1α) or siRNA-NC (as a negative control) using lipofectamine2000 (Invitrogen, Carlsbad, CA, United States). siRNAs were purchased from Biotend Co. Ltd. (Shanghai, China), the sequence of siRNA is as followed: F: UGAUACCAACAGUAACCAA; R: UUGGUUACUGUUGGUAUCA.

### CCK8 Cell Viability Assay

The CCK-8 assay kit (Cell Counting Kit-8; SAB, Shanghai, China) was utilized to determine the number of inhibited cells. 5000 SH-SY5Y cells were seeded into 96-well plates and 100 μl cell medium in each well. On the second day, cells were treated according to varying experiments. After a certain processing time, 10 μl of CCK-8 working solution was added to each well to incubate for about 2 h at 37°C. Furthermore, the wells containing only the culture medium served as blanks. The absorbance value at 450 nm of each well was measured using a microplate reader.

### Immunoblotting and Immunofluorescence Staining

All animal experiments were performed according to protocols approved by the Institutional Animal Care and Use Committee of Shanghai Institutes for Biological Sciences. After specific treatments, SH-SY5Y cells and dissected mouse brain tissues were lysed in cell or tissue lysis buffer.

We performed CO_2_ anesthesia and cardiac perfusion in 5 mice in each treatment group. One side of the striatum was isolated for Immunoblotting detection. Equal amount of the protein samples were first separated by SDS-PAGE and then transferred to PVDF membranes. Membranes were blocked with 5% non-fat milk in TBST for 1 h at room temperature and then the incubated overnight at 4°C with specific primary antibodies: anti-HIF-1α (#NB100-105, Novus), anti-AMPK (#5832, CST), anti-p-AMPK (#2535, CST), anti-TH (#T2928, Sigma). anti-LC3B (#2775S, CST), anti-p62 (ab91526, abcam), anti-Cathepsin D (#2284S, CST), anti-β-actin (#60008-1-lg), anti-Bax (#50599-2-lg), anti-Bcl-2 (#12789-1-AP), anti-HO-1 (#10701-1-AP), anti-SOD2 (#24127-1-AP), anti-Nrf2 (#16396-1-AP), and anti-PGC-1α(#66369-1-lg) were all purchased from Protein Tech Group. Then the PVDF membranes were rinsed with TBST for 3 times and 10 min each time. Membranes were incubated with the secondary antibody horseradish peroxidase conjugated at 1:5000 dilution in 5% non-fat milk/TBST for 1 h at room temperature. Then membranes were washed with TBST follow the previous method.

Mice brains were quickly removed and half of the mice brains were post fixed in 4% paraformaldehyde overnight at 4°C and immersed 30% sucrose solution at 4°C until sinking. Mice brain tissues were embedded with OCT, and were then cut into a series of coronal slices with a thickness of 30 μm. Brain sections were permeabilized and blocked with 10% goat serum for 1 h in room temperature, and then incubated with the following primary antibodies overnight at 4°C, finally incubated with the specific fluorescent secondary antibody for 1 h at room temperature. The intensity of each band was semi-quantified using Image J software.

### RNA Extraction and Analysis by Quantitative Real-Time PCR

SH-SY5Y cells after specific treatment were washed with PBS. Total RNA in cells was extracted using Trizol reagent (Invitrogen Life Technologies, Carlsbad, CA, United States) according to the manufacturer’s instructions. All RNA samples were purified and reverse transcribed into cDNA, and quantitative PCR analysis was performed as described (Dong et al., 2016). The primer sequences used were:

HIF-1α-F:GCGCGAACGACAAGAAA;
HIF-1α-R:GAAGTGGCAACTGATGAGCA;
18sRNA-F:CAGCCACCCGAGATTGAGCA;
18sRNA-R:TAGTAGCGACGGGCGGTGTG;
PGC-1α-F:AAACAGCAGCAGAGACAAATGC;
PGC-1α-R:TTGGTTTGGCTTGTAAGTGTTGTG.

### Annexin V-FITC/PI Assay

An Annexin V-FITC/PI kit (BD, Franklin Lakes, NJ, United States) was using to evaluate apoptosis according to the manufacturer’s instructions. SH-SY5Y cells were seeded into 6-well plates with 200000 cells in each well. After various treatment cells were harvested and washed twice with PBS. Annexin V-FITC and propidium iodide (PI) (each 4 μl) was added into Annexin V-FITC binding buffer (100 μl), and then added to the resuspended cells. After a gentle vortex, the cells were incubated with working solution for 30 min at room temperature in the dark. In the end, the number of cells which underwent apoptosis was counted by flow cytometry.

### Measurement of Mitochondrial Membrane Potential (MMP)

The MMP was determined by High-Content Screening (HCS). Each well of 48-well plates were seeded with 20000 SH-SY5Y cells. After specific treatments, cells were incubated in DMEM without serum containing 5 ng/μl hochest (Thermo scientific) at 37°C for 10 min without light. Then the cells were incubated in a 50 nM TMRM (Thermo scientific) 37°C for 30 min. Wash cells gently three times with DMEM, followed by HCS detection (Thermo scientific).

### Measurement of ATP Level

SH-SY5Y cells were harvested from 6 cm dishes after the treatments and the ATP Kit (Sigma) was utilized to determine the ATP level according to the manufacturer’s instructions. Briefly lysed with the assay buffer, followed by centrifugation at 12000 *g* for 10 min at 4°C. Finally, the level of ATP was measured by mixing appropriate supernatant with reaction mix to each of the wells. Mix well using a horizontal shaker. Incubate the plate at 37°C for 30 min. And protect the plate from light during the incubation. Measure the absorbance at 570 nm. Using the corrected measurement, determine the amount of ATP present in the sample from the standard curve.

### Measurement of Mitochondrial Respiratory Capacity

To study OCR, SH-SY5Y cells were seeded into Seahorse 24-well plate with 8000 cells in each well and with specific treatments for 24 h according to previous study (Mäkelä et al., 2016). OCR was determined by the Seahorse XFe24 analyzer (Seahorse Bioscience, Boston, MA, United States) and using the Mitostress kit (Seahorse Bioscience) employing 1 μM of Oligomycin and rotenone, 500 nM of carbonylcyanide 4-(trifluoromethoxy) phenylhydrazone (FCCP) as manufacturer’s instructions.

### Reactive Oxygen Species (ROS) Measurement

On the day of study, the cells in 10-cm dishes were rinsed three times with 3 ml of chilled PBS buffer and then exposed to 25 μM dihydroethidium (DHE) for 20 min at 37°C in PBS buffer containing 0.1% DMSO. DHE was then washed from the cells to avoid absorption of any extracellular oxyethidium formed by autoxidation of DHE. The cells were then harvested by scraping and were placed in 300 μl of cold methanol to homogenized, and followed by centrifugation at 14000 *g* for 5 min at 4°C. About 40 μl of the supernatant were used for BCA Protein Assay. HPLC was selected to detect the content of ROS. The supernatant of the cells (20 μl) was injected directly into the column.

### Animal Preparation and Drug Administration

We purchased several male C57BL/6 mice (10–12 weeks, 22–26 g) from the Shanghai SLAC Laboratory Animal Company (Shanghai, China). Following acclimatization, the animals were randomized into three groups: CON, MPTP, and MPTP + FG-4592 (each group *n* = 10). MPTP (30 mg/kg/day) and FG-4592 (10 mg/kg/day) were injected intraperitoneally (i.p.) for 5 consecutive days. FG-4592 was pretreated 6h before intraperitoneal injection of MPTP. MPTP (M0896, Sigma) was dissolved in normal saline, stock concentration at 3 mg/ml. FG-4592 (S1007, Selleck) was suspended in DMSO, stock concentration at 50 mg/ml and it was diluted to 1 mg/ml with normal saline before use. CON group also injected equal concentration of DMSO. All animals were pretrained for each behavioral test and mice with abnormal performance were excluded. Behavioral tests were conducted 2 days after the final MPTP injection.

### Open Field Test and Pole Test

Open field test was performed in a quiet environment. Each mouse was placed in the center of the bottom of the metal box (open field: 80 × 80 × 28.5 cm). Clean the inner wall and the bottom of the box to prevent the remaining information of the last animal (such as animal’s urine, urine smell) affect the next test results. Change animals and keep experimenting. The activity of mice was monitored for 15 min.

The pole test was performed according to previously published methods (Drucker-Colin and Garcia-Hernandez, 1991). Firstly, a 55 cm high and 10 mm diameter vertical pole was required. The pole was surrounded by a rough material like fabric and then placed in the home cage. Mice were placed near the top of the pole, with their heads up, and the total time to climb down was recorded.

### HPLC Determination of Dopamine and Its Metabolites in Mice

Stripped striatum is weighed, and 10 μl/mg PBS containing 10 mM EDTA is added. After being on ice for 15 min, the tissues were sonicated and centrifuged at 15000 rpm for 10 min at 4°C. Collected the supernatant and calculated the supernatant volume for each sample. Then the supernatant was added to an equal volume of ice-cold 0.4 M perchloric acid containing 10 mM EDTA and vortexed. After standing on ice in the dark for 15 min, the medium was centrifuged at 15000 rpm for 15 min at 4°C. The middle layer was used to detect the levels of dopamine (DA), dihydroxypheny lacetic acid (DOPAC), and HVA. The supernatant of the striatal tissue (20 μl) was injected directly into the column and detected as described in our previously described method (Beal et al., 1992).

### Statistical Analysis

Each experiment was performed at least three times include biological and technical replicates. Data were presented as mean ± SD, and the differences were evaluated using *t*-tests or two-way ANOVA using GraphPad Prism software version 5. *P*-values less than 0.05 were considered statistically significant.

## Results

### MPP^+^ Stimulated the Proliferation Inhibition, Apoptosis and Influenced the Expression Level of HIF-1α in SH-SY5Y Cells

**FIGURE 1:**
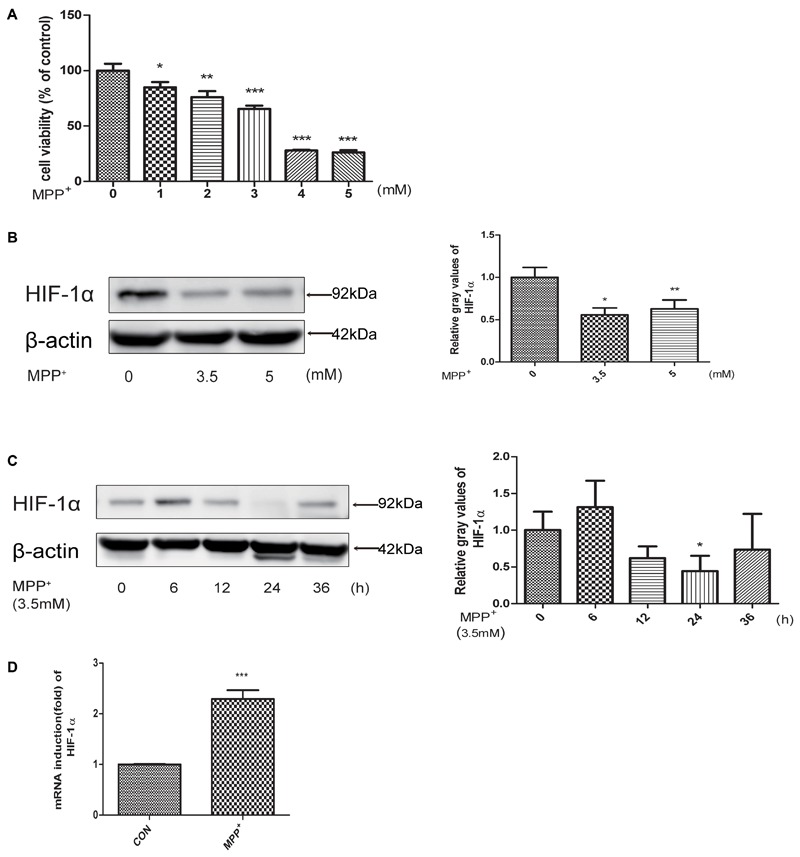
MPP^+^ stimulated the proliferation inhibition, apoptosis and decreased the expression level of HIF-1α. **(A)** SH-SY5Y cells were treated with MPP^+^ for 24 h at various concentrations. And then examined the proliferation inhibition rates by CCK-8 method. Western blot and quantification of HIF-1α protein after MPP^+^ treatment for various concentrations **(B)** or at 3.5 mM for different time periods **(C)**. qRT-PCR assay was used to test the changes of HIF-1α at mRNA level after treated with MPP^+^ for 24 h at 3.5 mM **(D)**. Data are expressed as mean ± SD. ^∗^*P* < 0.05, ^∗∗^*P* < 0.01, ^∗∗∗^*P* < 0.001(*n* ≥ 3), compared to the control.

To evaluate the neurotoxicity of MPP^+^ in SH-SY5Y cells, we first treated cells with MPP^+^ at various concentrations (0, 1, 2, 3, 4, 5 mM) for 24 h, and then examined the cell inhibition rate by CCK-8 method. We found that cells treated with MPP^+^ at a concentration of 3.5 mM achieved an approximate 50% inhibition rate of cell proliferation (**Figure 1A**). So we chose 3.5 mM for subsequent cell treatments. Then we examined the protein level of HIF-1α in SH-SY5Y cells at different MPP^+^ concentrations (0, 3.5, 5 mM) after 24 h and 3.5 mM for different time periods (0, 6, 12, 24, 36 h). As shown in **Figures 1B,C**, the protein levels of HIF-1α was significantly reduced in different dose and time course. However, the mRNA expression of HIF-1α was increased (**Figure 1D**), indicating that mitochondrial inhibitors may affect the degradation of HIF-1α, rather than affecting its gene expression.

### FG-4592 Increased the Expression of HIF-1α and Attenuated MPP^+^ Induced Cell Damage in SH-SY5Y Cells

**FIGURE 2:**
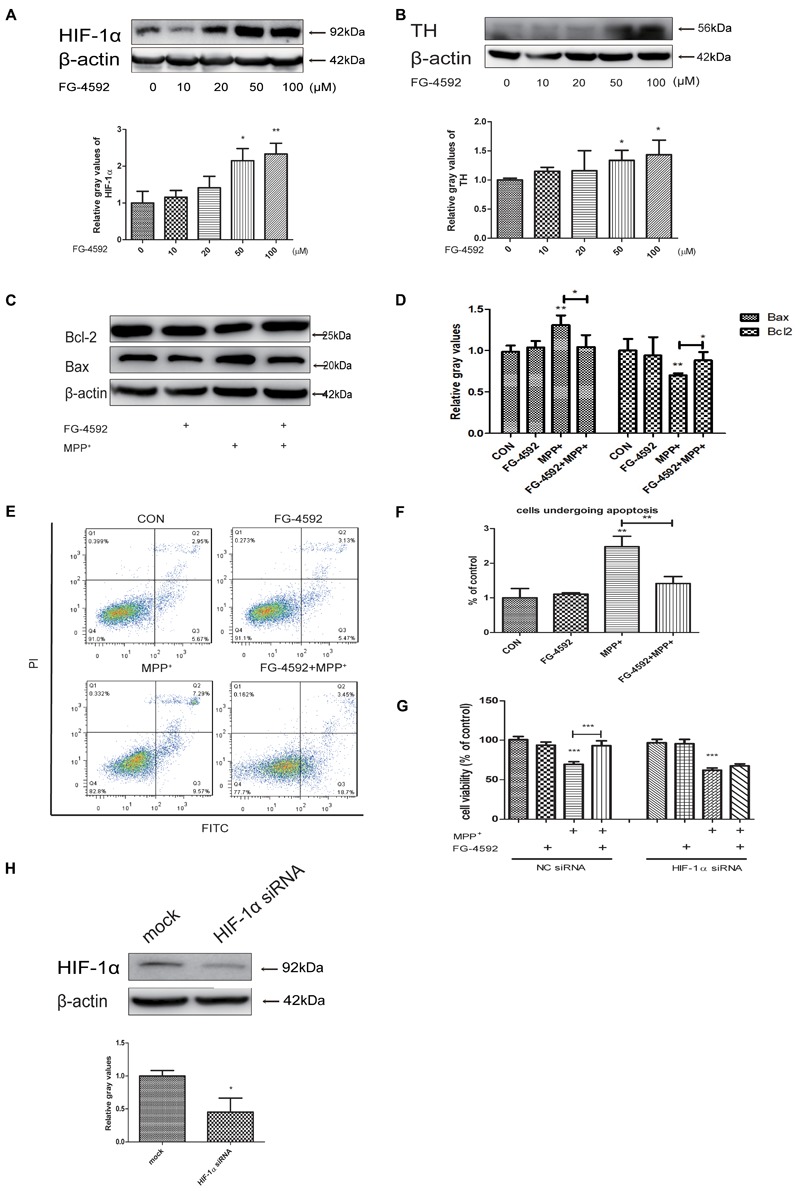
FG-4592 increased the expression of HIF-1α and attenuated MPP^+^-induced cell damage in SH-SY5Y cells. **(A)** SH-SY5Y cells were treated with FG-4592 at various concentrations for 24 h. **(B)** Cells were treated with FG-4592 at 50 μM for different time durations. SH-SY5Y cells were exposed to MPP^+^ (3.5 mM) in the presence or absence of FG-4592 for 24 h, apoptosis of cells was determined by immunoblotting assay **(C,D)** and Annexin V-FITC assay **(E,F)**, Cell viability was determined by CCK8 assay **(G)**. **(H)** Represented the protein level in SH-SY5Y cells after transfected with siRNA of HIF-1α. The quantification of HIF-1α protein was as below. Data were expressed as means ± SD, ^∗^*P* < 0.05, ^∗∗^*P* < 0.01, ^∗∗∗^*P* < 0.001 as compared to the control or MPP^+^(*n* ≥ 3).

FG-4592 can cause the accumulation of HIF-1α through inhibition of its degradation and previous studies showed a direct linkage between HIF-1α and TH, which is the rate-limiting enzyme in the synthesis of DA in dopaminergic neurons (Millhorn et al., 1997; Haavik and Toska, 1998; Schnell et al., 2003). Our experiments found that FG-4592 caused a dose-dependent increase of HIF-1α, accompanied by the induction of TH (**Figures 2A,B**). We then examined the apoptosis of SH-SY5Y after pretreatment with FG-4592. Confirmed by immunoblotting assay, the MPP^+^-induced increases of Bax and decrease of Bcl-2 protein levels in SH-SY5Y cells were partially reversed by FG-4592 pre-treatment (**Figures 2C,D**). The Annexin V-FITC/PI assay showed that apoptosis caused by MPP^+^ was also drastically reduced when co-treated with FG-4592 at 24 h (**Figures 2E,F**). To further explore the role of HIF-1α in the neuroprotection exhibited by FG-4592, we used HIF-1α siRNA for reduction of HIF-1α in SH-SY5Y cells. The control group were cells transfected with NC siRNA. After 48 h siRNA transfection, we performed specific drug treatment to cells. The toxic effect of MPP^+^ was reversed when cells were pre-treated with FG-4592 in CCK8 assay. Nevertheless, there was no such reversal effect in FG-4592 pre-treated HIF-1α siRNA group (**Figure 2G**). **Figure 2H** represented the protein level in SH-SY5Y cells after treated with HIF-1α siRNA. Taken together, these observations support the assumption that the FG-4592 could protect SH-SY5Y cells from the MPP^+^ induced cell death and apoptosis. In addition, these neuroprotective effect of FG-4592 could be mediated, at least in part, by HIF-1α induction.

### FG-4592 Increased Mitochondrial Biogenesis and Respiration in Dopaminergic Neurons

**FIGURE 3:**
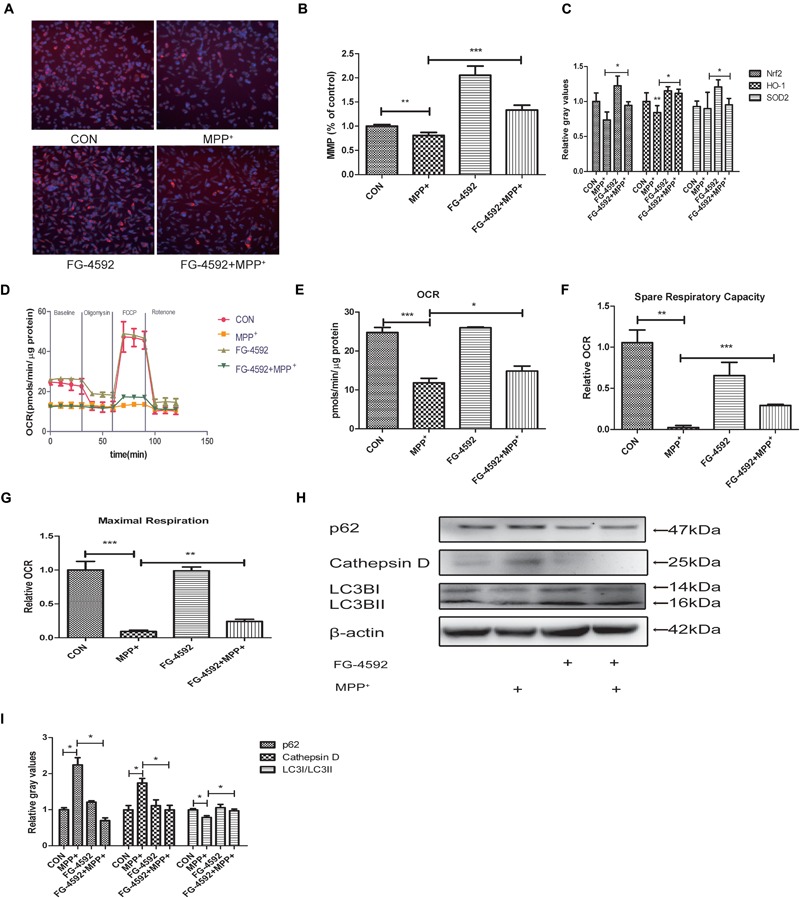
Mitochondrial biogenesis and respiration are increased in dopaminergic neurons treated with FG-4592. SH-SY5Y cells were exposed to MPP^+^ (3.5 mM) in the presence or absence of FG-4592 (50 μM) for 24 h. Mitochondrial membrane potential (MMP) in cells was measured using the HCS **(A,B)**, red represents MMP and blue represents nuclei. ATP production **(C)** was detected by commercial kits as described in Methods. Mitochondrial oxygen consumption rate (OCR) in human dopaminergic neurons was measured using the Seahorse XFe24 analyzer as described **(D)**. We also list the OCR quantification **(E)**, Spare respiratory capacity **(F)** and Maximal respiration **(G)**. Western blotting analysis and quantification of the relative p62, Cathepsin D, LC3BI and LC3BII protein abundance in the cells were showed in **(H,I)**. β-protein served as the control. Data were expressed as means ± SD, ^∗^*P* < 0.05, ^∗∗^*P* < 0.01, ^∗∗∗^*P* < 0.001 as compared to the control or MPP^+^ (*n* = 3).

In 1979, it was observed that Parkinsonism could be induced by a toxin which could inhibit mitochondrial respiratory complex I. This initiated the hypothesis that mitochondrial dysfunction may play a vital role in the pathogenesis of PD (Davis et al., 1979; Langston et al., 1983). We hypothesized that FG-4592 may protect cells by rescuing mitochondria dysfunction. We measured the MMP in SH-SY5Y cells co-treated with FG-4592 and MPP^+^. The results showed that the increase of HIF-1α could partially reverse the MPP^+^ reduction of MMP (**Figures 3A,B**). In order to determine whether increased mitochondrial MMP was accompanied by functional changes in cellular metabolism, ATP levels (**Figure 3C**) and a real-time analysis of mitochondrial respiration (**Figure 3D**) in the different conditions were compared. Results showed that particularly the oxygen consumption rate (OCR) (**Figure 3E**), spare respiration capacity (**Figure 3F**) and maximal respiration (**Figure 3G**) in these neurons were increased by FG-4592 pre-treatment compared with cells treated with MPP^+^ alone.

Emerging findings suggest that the autophagy-lysosome pathway is also compromised in PD (Gao et al., 2017). Since mitochondrial clearance depends mainly on the autophagy pathway, autophagy is important for mitochondrial quality control. Poor quality mitochondria may enhance cellular oxidative stress, generate apoptosis signals, and induce cell death (Zhang, 2013). Therefore, interventions that stimulate mitophagy to maintain mitochondrial function is expected to be an effective approach to delay the neurodegenerative processes in PD. We next detected the level of autophagy in SH-SY5Y cells. Large polyubiquitinated protein aggregates are often tend to be degraded in autophagosomes, such as p62 and Cathepsin D (Lamark and Johansen, 2012; Aufschnaiter et al., 2017). Protein aggregation have thus been a hallmark of neurodegenerative disease including PD (Masters and O’Neill, 2011). Our results showed that the expression of p62 and Cathepsin D were increased after treatment with MPP^+^ while the ratio of LC3II/LC3I was decreased (**Figures 3H,I**). When we pre-treated cells with FG-4592, the inhibition of autophagy was alleviated.

### FG-4592 Prevented the MPP^+^ Induced Generation of ROS and Increased the Expression of Antioxidant Genes

Many studies suggested that mitochondrial dysfunction and oxidative stress played a crucial role in PD pathogenesis (Fujita et al., 2011). Dopaminergic neurons are susceptible to oxidative damage due to high levels of inherent ROS that are produced during DA synthesis or its breakdown by monoamine oxidases (Cohen et al., 1997; Haavik, 1997).

**FIGURE 4:**
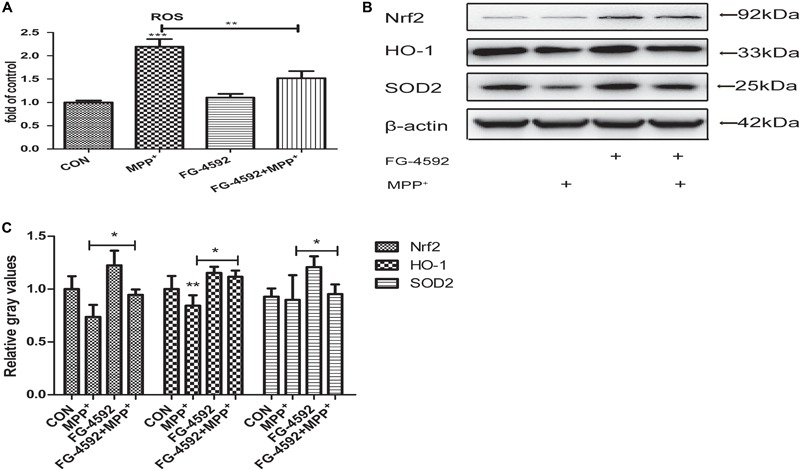
FG-4592 prevented the MPP^+^ induced generation of reactive oxygen species (ROS and increased the expression of antioxidant genes). SH-SY5Y cells were exposed to MPP^+^ (3.5 mM) in the presence or absence of FG-4592 (50 μM) for 24 h. The ROS level in different treated cell groups was showed in **(A)**. The protein level of Nrf2, HO-1 and SOD2 were determined by immunoblotting assay **(B)**. Western blotting analysis and quantification of the relative Nrf2, HO-1 and SOD2 protein abundance in the cells were showed in **(C)**. β-protein served as the control. Data were expressed as mean ± SD, ^∗^*P* < 0.05, ^∗∗^*P* < 0.01, ^∗∗∗^*P* < 0.001 as compared to the control or MPP^+^ (*n* = 3).

We explored the impact of HIF-1a activation through FG-4592 upon the generation of ROS during the MPP^+^ treatment. ROS production was increased significantly in cells that had been treated with MPP^+^. When cells were pre-treated with FG-4592, this ROS generation was significantly suppressed (**Figure 4A**). It has been shown that the expression of SOD2, Nrf2 and HO-1 which mediate ROS detoxification is an important defensive mechanism against oxidative stress. Western blots revealed that the expression of these three genes were increased when treated with FG-4592 alone or in cells pre-treated with FG-4592 in comparison to control cells or cells treated with MPP^+^(**Figures 4B,C**). Thus, upregulation of antioxidant enzymes and decreased ROS production seems to be important consequences of HIF-1a activation and may contribute to the protection of dopaminergic neurons from the cytotoxic effects of MPP^+^.

### FG-4592 Increases the Expression of PGC-1α in Human Dopaminergic Neurons

**FIGURE 5:**
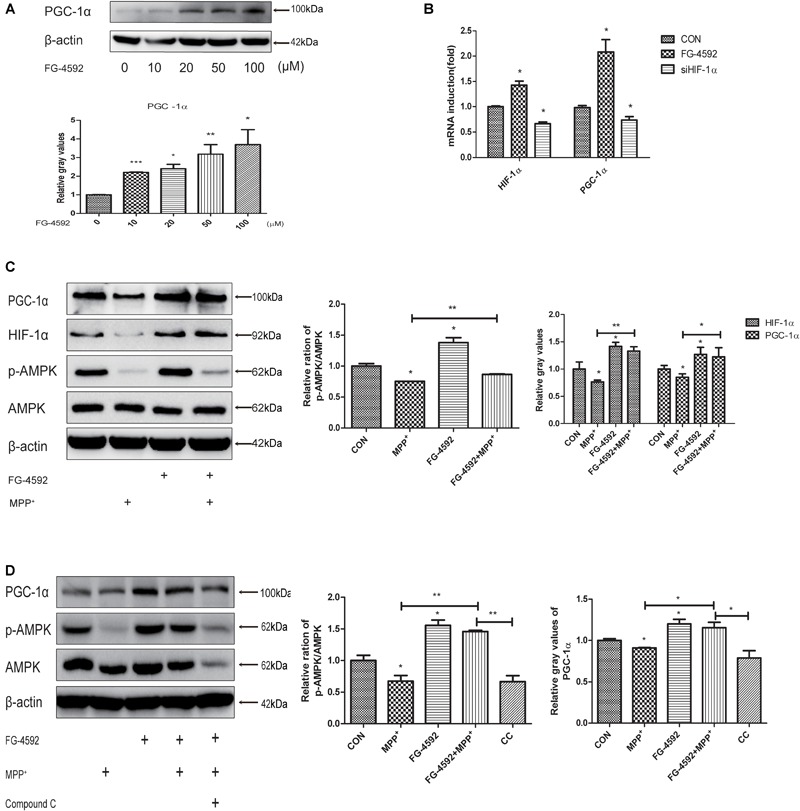
FG-4592 increases the expression of PGC-1α in human dopaminergic neurons. SH-SY5Y cells were treated with FG-4592 at various concentrations for 24 h. The protein level of PGC-1α were determined by immunoblotting assay **(A)**. The mRNA level of PGC-1α were determined by qRT-PCR **(B)**. Then SH-SY5Y cells were exposed to MPP^+^ (3.5 mM) in the presence or absence of FG-4592 (50 μM). The protein level of PGC-1α, p-AMPK and AMPK were determined by immunoblotting assay **(C)**. SH-SY5Y cells were exposed to MPP^+^ (3.5 mM) in the presence or absence of FG-4592 (50 μM) and Compound C (10 μM) for 24 h. Western blotting analysis and quantification of the relative PGC-1α, p-AMPK and AMPK protein abundance in the cells. β-protein served as the control **(D)**. CC represents cells that were co-treated with MPP^+^, FG-4592 and Compound C. Data were expressed as mean ± SD, ^∗^*P* < 0.05, ^∗∗^*P* < 0.01, ^∗∗∗^*P* < 0.001 as compared to the control or MPP^+^ (*n* = 3).

Peroxisome proliferator-activated receptor-γ coactivator-1α is a transcriptional co-activator that regulates mitochondrial biogenesis and respiration as well as the cell defense system against ROS (Wu et al., 1999; Puigserver and Spiegelman, 2003; St-Pierre et al., 2006). AMPK, an enzyme sensor, is always activated upon energy depletion in muscle (Steinberg and Kemp, 2009). AMPK phosphorylates PGC-1α on specific serine and threonine residues and this results in increased mitochondrial gene expression (Scarpulla, 2011). PGC-1α showed protective effects in different brain diseases models (Mudò et al., 2012). To investigate whether expression of PGC-1α was altered in SH-SY5Y cells with different treatments, we performed quantitative PCR and immunoblotting assay and found that addition of FG-4592 increased the PGC-1α protein (**Figure 5A**) and mRNA (**Figure 5B**) levels in SH-SY5Y cells as well as p-AMPK and AMPK (**Figure 5C**). The p-AMPK inhibitor compound C decreased p-AMPK levels in the neurons as well as partially inhibited the increase of PGC-1α brought about by FG-4592 (**Figure 5D**). Taken together, FG-4592 can increase the expression of PGC-1α, and AMPK-PGC-1α in human dopaminergic neurons.

### FG-4592 Improved the Behavioral Impairments of MPTP-Treated Mice

**FIGURE 6:**
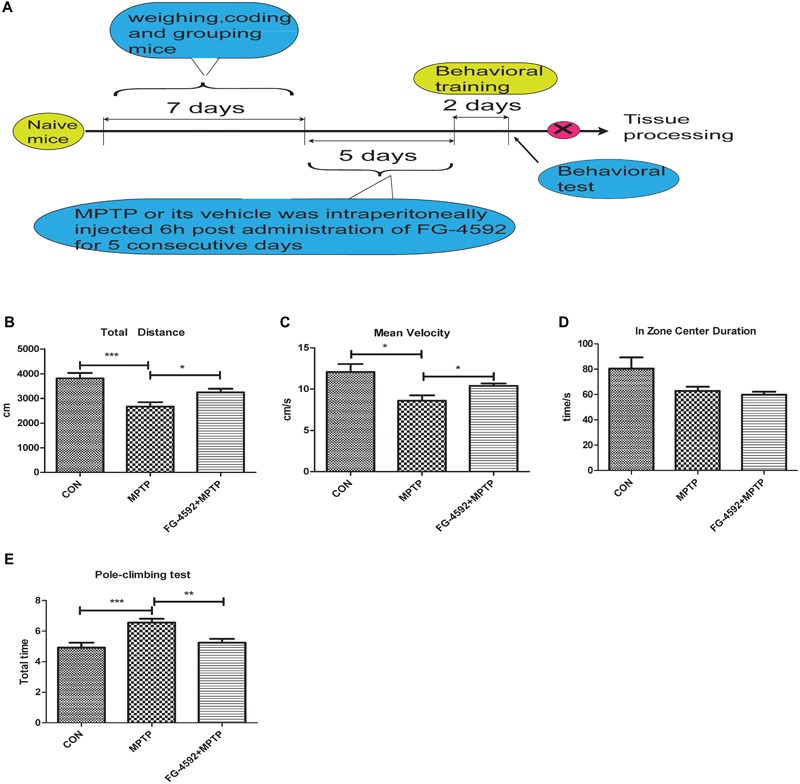
FG-4592 reverses the behavioral impairments of MPTP-treated mice. A timeline detailing the coding, grouping, behavioral training, and time points for behavioral tests as well as drug treatments and tissue processing **(A)**. We studied the locomotor activity in MPTP-treated mice by open field test, and list the total distance, mean velocity and zone center duration **(B–D)**. We further evaluated MPTP-induced bradykinesia and coordination in mice by the pole test and list the total time in pole-climbing test **(E)**. Data were expressed as means ± SD, ^∗^*P* < 0.05, ^∗∗^*P* < 0.01, ^∗∗∗^*P* < 0.001 as compared to control or MPTP treated group (each group *n* = 10).

We next used a mouse model of PD to test the effectiveness of FG-4592 by studying the locomotor activity in MPTP-treated mice by open field test. The results demonstrated that there was a significant reduction in total distance, mean velocity and zone center duration in MPTP-treated mice. Among them, zone center duration can reflect the anxiety of mice to a certain extent. Administration of FG-4592 (**Figure 6A**) significantly rescued these reductions in locomotor activities in MPTP-treated mice, mainly reflected in significant reduction in total distance (**Figure 6B**), mean velocity (**Figure 6C**). However, it failed to improve their anxiety states according to the results of zone center duration (**Figure 6D**).

Next step, pole test was chose to evaluated MPTP-induced bradykinesia and coordination in mice. We discovered that the total time was significantly prolonged after MPTP treatment. Administration of FG-4592 significantly reversed the – prolongation of total time induced by MPTP in the pole test (**Figure 6E**). To sum up, our results suggest that FG-4592 can partially improve the behavioral impairments induced by MPTP.

### FG-4592 Rescued the Depletion of DA and TH Protein in the Striatum, and the Loss of TH Positive Neurons in the Substantia Nigral Induced by MPTP

**FIGURE 7:**
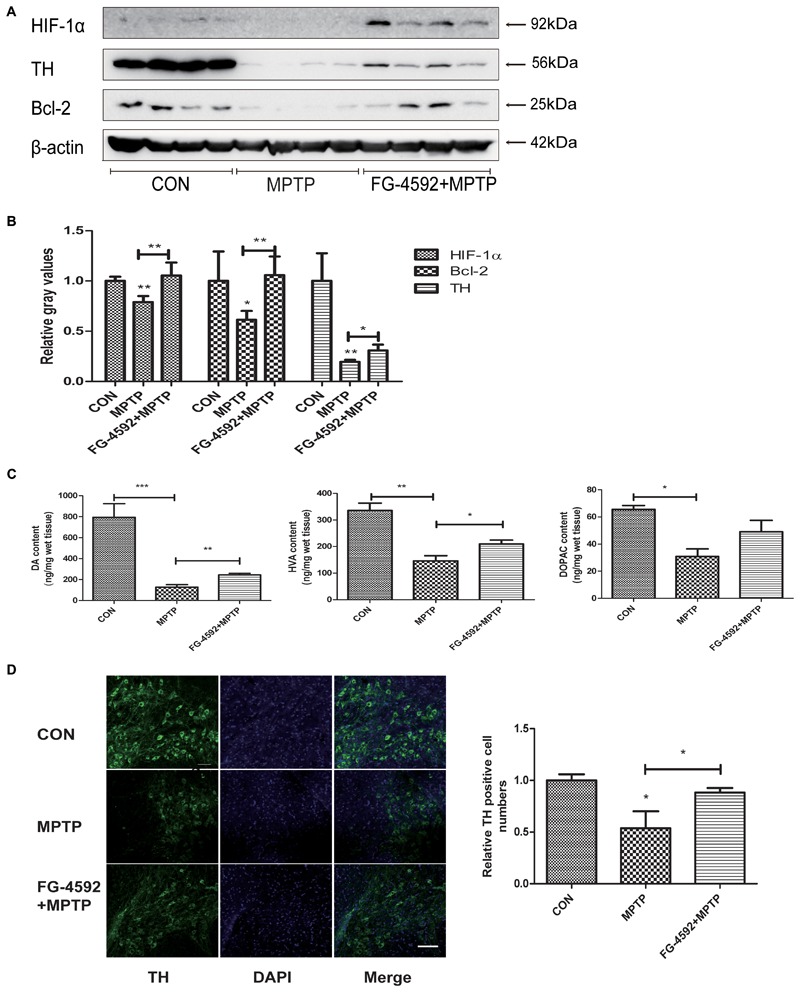
FG-4592 rescued the depletion of DA and TH protein in the striatum, and the loss of TH positive neurons in the substantia nigral induced by MPTP. Western blotting analysis and quantification of relative TH, HIF-1α and Bcl-2 protein abundance in the striatum **(A,B)**. β-protein served as the internal control. The levels of DA, DOPAC, and HVA **(C)** were calculated and expressed as nomogram per milligram wet tissue weight. Immunofluorescence images of TH (green) were taken in the SN. Nuclei were revealed by DAPI staining (blue), with the merged images depicted at the right **(D)**. Scale bars: 100 μm. Data were expressed as mean ± SD (*n* = 5). ^∗^*P* < 0.05, ^∗∗^*P* < 0.01, ^∗∗∗^*P* < 0.001 as compared to the control or MPTP treated group.

We first used Western blotting to examine the level of the striatal TH protein in mice. Consistent with the above results, TH protein level increased significantly in the FG-4592+MPTP group compared with the MPTP-treated mice. In addition, **Figure 7A** showed that FG-4592 suppressed the decrease of HIF-1α and Bcl-2, quantitative data were showed in **Figure 7B**.

In order to investigate whether FG-4592 affected the levels of neurotransmitters, we used HPLC as the testing method. After treated with MPTP, the levels of DA, DOPAC, and HVA were significantly reduced in mice striatal (**Figure 7C**). However, the reduction of DA and its metabolites induced by MPTP was significantly attenuated by administration of FG-4592. These results told us that FG-4592 might both affect the synthesis and catabolism of DA.

Next, we used immunofluorescence staining to detect TH-positive neurons in MPTP-treated mice and observed that the dopaminergic neurons were decreased as shown in **Figure 7D**. Treatment with FG-4592 increased the number of substantia nigral TH positive neurons compared with the MPTP group.

## Discussion

The role of HIF in the pathogenesis of PD is well documented by our previous study and other groups; but the therapeutic potential of this pathway remains poorly explored. In a hypoxic environment, the HIF acts as a transcription factor triggers a wide range of coordinated responses in numerous tissues, not only erythropoiesis and angiogenesis but also cytoprotection, including cardioprotection, renoprotection and neuroprotection(Bouchie, 2013). HIF heterodimer consisting of a constitutively expressed β subunit and an oxygen-regulated α subunit (Hackenbeck et al., 2011). These two subunits are constantly made in nearly all cell in the body, but the function of HIF is kept in check by degradation after hydroxylation by PHD under normal oxygen conditions. Under hypoxic conditions, the degradation of HIF-1α is attenuated because of HIF-PH requires oxygen as a substrate, which leads to the accumulation of HIF-1α. Initially found to be related to HIF-1α in PD is TH, as TH is increased by the induction of HIF-1α (Schnell et al., 2003). Many studies have demonstrated that HIF-PHI can reduce cell death in ischemic stroke and PD, mainly including some iron chelator, like 3, 4-DHB, compound A, DMOG and DFO. As to chronic neurodegenerative conditions like PD, the daily use of an HIF-PHI that chelates iron may result in restless legs syndrome (RLS) (Earley et al., 2014) or anemia (White, 2005), therefore, the use of an HIF-PHI that not targets iron might has potential advantages.

FG-4592 is a short half-life oral HIF-PHI, along with an intermittent dosing regimen, gives rise to transient increase of HIF activity (Besarab et al., 2016). Unlike other selective chemical tools, peptide inhibitors, and short interfering RNAs, as it is currently in phase 3 trials to treat anemia in patients with CKD (Bouchie, 2013), it has better clinical application prospects. It triggers the body’s natural response to hypoxia and also is found protecting against spinal cord injury (Wu et al., 2016). Whereas, little is known about the specific role of FG-4592 in PD. In our study, it was firstly demonstrated that FG-4592 could attenuate MPP^+^ induced decreased cell viability and increased apoptotic cells, indicating that MPP^+^ induced SH-SY5Y cell injury was significantly attenuated by administration of FG-4592. However, how FG-4592 provides neuroprotection under the background of PD remains to be elucidated.

The present study introduces HIF-1α can abolish mitochondrial impairment (Hwang et al., 2015; Neitemeier et al., 2016). Mitochondrial dysfunction also plays a crucial role in the pathogenesis of PD. Therefore, we wanted to explore whether FG-4592 can achieve neuroprotective effects by protecting mitochondrial function. Our studies confirmed that FG-4592 could restore mitochondrial function, mainly reflect in increased MMP, ATP production and mitochondria respiration capacity. On the other hand, autophagic removal of mitochondria is important for mitochondrial quality control (Yoo and Jung, 2018). Moreover, both mitochondrial and autophagy-lysosomal dysfunction have been found in postmortem PD brains (Ryan et al., 2015) as well as dopaminergic neurons derived from patients with idiopathic and familial PD (Burbulla et al., 2017; Zhang et al., 2018). Considering the close relationship between autophagy pathway and mitochondrial function, the expression of autophagy-related proteins was examined. We found that FG-4592 also induces autophagy in SH-SY5Y cells.

Oxidative stress as one of the important consequences of mitochondrial dysfunction and impaired autophagy dysfunction, is linked to neurodegenerative diseases, such as PD (Henchcliffe and Beal, 2008). Similarly, improved mitochondrial function and increased clearance of damaged mitochondria may reduce the oxidative stress in cells. We here studied the impact of FG-4592 on ROS level. Data show that treatment with FG-4592 is able to decrease the production of ROS caused by MPP^+^. It also induces some anti-oxidant enzymes including Nrf2, HO-1 and SOD2. Whether it is to improve mitochondrial function or increased the expression of anti-oxidant enzymes can achieve the protective effect on SH-SY5Y cells.

In order to investigate the molecular regulation of mitochondrial function by HIF-1α, we examined the expression of PGC-1α, a key regulator of mitochondrial biogenesis and respiration, and found that there is a positive correlation between them. Recently, some studies have shown that transgenic mice overexpressing PGC-1α in dopaminergic neurons are resistant against cell degeneration induced by the neurotoxin (MPTP) (Mudò et al., 2012). Conversely, an increased vulnerability to MPTP induced degeneration of substantia nigral dopaminergic neurons was observed in PGC-1α knockout mice (St-Pierre et al., 2006). Despite extensive effort on studying the function of PGC-1α in mitochondria, PGC-1α also reduces α-synuclein oligomerization and rescues α-synuclein mediated toxicity (Eschbach et al., 2015). Therefore, we hypothesize that impairment of PGC-1α mediated mitochondria regulation could contribute to the pathogenesis of PD neuropathy. PGC-1α has some upstream regulators, such as NAD-dependent deacetylase sirtuin-1 (SIRT1) and AMPK (Zheng et al., 2010). In our current and previous studies we found that the expression of p-AMPK is dramatically decrease in MPP^+^ (or Rotenone) treated SH-SY5Y cells (Wu et al., 2011), and p-AMPK inhibitor compound C also inhibits the increase in PGC-1α brings about by FG-4592. In conclusion, the up-regulation of PGC-1α caused by FG-4592 is achieved at least in part by upregulating the level of p-AMPK.

**FIGURE 8:**
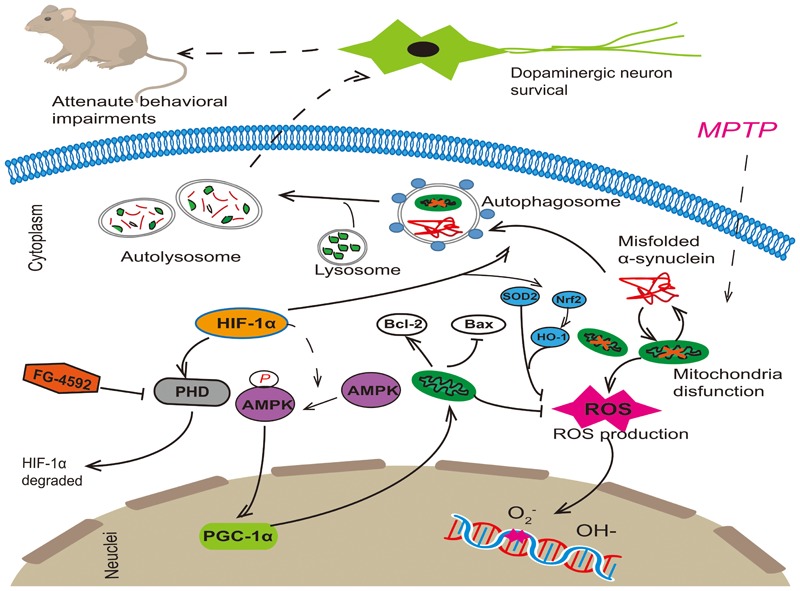
FG-4592 activated HIF-1α by inhibiting PHD and decrease its degradation. Activated HIF-1α could increase the protein level of p-AMPK and then induced the expression of PGC-1α. It also enhanced autophagy, which can remove damaged mitochondria and misfolded proteins in Dopaminergic neurons. This promoted the function of mitochondria, reduced cell apoptosis and decreased the production of ROS, which rescued the MPTP-induced loss of dopaminergic neurons, finally attenuating the behavioral impairments of MPTP-treated mice.

As we all know, mitochondria dysfunction, decreased clearance of poor quality mitochondria and oxidative stress all make important contribution to pathogenesis of PD, and there is a close interrelationship among them. Mitochondria are major core in the cell integrating energy demand and reactive species. Oxidative stress can either be a signal to activate autophagy, or exert damage to the autophagy machinery to inhibit autophagy (Feng et al., 2005; Scherz-Shouval et al., 2007). Reciprocally, autophagy may decrease cellular oxidative stress by clearance of toxic proteins and damaged mitochondria or decrease specific antioxidants (Yu et al., 2006). A similar relationship between mitochondrial activities and autophagy also exists. Research showed that mitochondria-deficient cells exhibit attenuated induce of autophagic gene and autophagic flux in response to starvation (Graef and Nunnari, 2011). Take above reasons into consideration, we think that mitochondrial function, autophagy and oxidative stress all play an important role in the development of PD (**Figure 8**). The interaction between them is complicated and tight. If we want to achieve neuroprotection effect on dopaminergic cells, any one of the three cannot be ignored. Therefore, FG-4592 as a new HIF-PHI may provide great potential therapeutic benefits for patients with PD.

## Conclusion

In our study, we demonstrate that FG-4592 could attenuate MPP^+^ induced decreases in cell viability, cell autophagy, mitochondria function and increases in oxidative stress. The underlying mechanism may include the activation of AMPK-PGC-1α pathway. Our results also indicate that FG-4592 could rescue the loss of dopaminergic neurons in MPTP-treated mice, and finally attenuate the behavioral impairments of them. Therefore, FG-4592 as a new HIF-PHI may be a potential therapeutic for patients with PD.

## Author Contributions

Y-CW and HY provided fund support, revised the manuscript, and designed the project ideas. HX revised the manuscript. XL conducted the experiments and wrote the manuscript. X-XC, Y-JC, and T-TW provided the experimental materials and assist XL in problem-solving.

## Conflict of Interest Statement

The authors declare that the research was conducted in the absence of any commercial or financial relationships that could be construed as a potential conflict of interest.

## Abbreviations

AMPK: AMP-activated protein kinase
Bax: Bcl-2-associated X protein
Bcl-2: B-cell lymphoma 2
CKD: chronic kidney diseases
DA: dopamine
DOPAC: dihydroxyphenylacetic acid
HIF-1α: Hypoxia-inducible factor-1α
HIF-PHI: HIF PHD inhibitor
HO-1: heme oxygenase-1
HVA: homovanillic acid
MMP: mitochondrial membrane potential
MnSOD: manganese superoxide dismutase
MPP+: 1-methyl-4-phenyl-pyridinium ion
MPTP: 1-methyl-4-phenyl-1,2,3,6-tetrahydropyridine
Nrf-2: nuclear factor erythroid 2 p45-related factor 2
OCR: mitochondrial oxygen consumption rate
PGC-1α: Peroxisome proliferator-activated receptor-γ coactivator-1α
PHD: prolyl hydroxylases
pVHL: von Hippel-Lindau
ROS: reactive oxygen species
SOD2: superoxide dismutase 2
TH: tyrosine hydroxylase
